# 989 Complications Following Laser Resurfacing of Hypertrophic Burn Scars – a Single Center Experience

**DOI:** 10.1093/jbcr/iraf019.520

**Published:** 2025-04-01

**Authors:** Raphaella Lambert, Sebastian Vrouwe

**Affiliations:** Pritzker School of Medicine, University of Chicago; University of Chicago Burn Center

## Abstract

**Introduction:**

Hypertrophic scars are a leading cause of morbidity in burn survivors, contributing to both functional and psychosocial challenges. Laser resurfacing, specifically the ablative fractional CO2 laser (AFL-CO2), is a promising modality in managing the various sequelae of hypertrophic burn scars including pruritis, erythema, stiffness, and tightness. Despite growing demand for laser resurfacing in burn patients, there are limited reports that highlight the actual peri- and post-operative complication rates following this procedure. Furthermore, there is no standardized protocol on how to utilize the laser in burn scars; therefore, reporting from different programs is necessary to confirm the safety of this technology. Here, we describe our center’s early experience with ablative fractional laser resurfacing in the management of hypertrophic burn scars during the initial three years of the program.

**Methods:**

We retrospectively reviewed all patients who underwent AFL-CO2 for functional improvement of hypertrophic scars secondary to burn injury at an academic medical center between March 1, 2021 to February 29, 2024. A minimum of 6 weeks of follow-up was necessary to track peri-operative and post-operative complications. Data was analysed using Microsoft Excel and R.

**Results:**

A total of 110 sessions across 40 patients (18 adult, 22 pediatric) were included for retrospective review. The average age at treatment was 27.3 years, with 20 male and 20 female patients. The most common injury etiologies were flame (40%) and scald (32.5%) burns, with an average 13.8% total body surface area affected. The average time from injury to first laser treatment was 46.6 months (range 6-765). A mean 2.75 cases were performed per patient during the study period (range 1-5). All cases were performed in the operating room, with the majority utilizing general anesthesia (106/110, 96.5%). An average area of 229.4 sq cm and 6.5% TBSA was treated in adults and children, respectively. The same AFL-CO2 platform was used for all cases, using the deep mode with a mean maximum energy of 45 mJ and 5% density with a single pass. No infectious complications, skin complications, unplanned admissions or emergency department visits were observed in the 6 weeks following any laser treatments; 2 (1.8%) patients experienced laryngospasm or bronchospasm on extubation.

**Conclusions:**

AFL-CO2 is a safe and well-tolerated option for the management of hypertrophic burn scars. Further research is warranted to further explore the efficacy of this approach in the management of hypertrophic burn scars.

**Applicability of Research to Practice:**

Our research demonstrates the safety profile of AFL-CO2 in burn scar management.

**Funding for the Study:**

N/A

